# Supplementary material for: Optimising housing typology distributions for multi-hazard loss reductions in resource-constrained settings
Source: Commun Eng. 2025 Oct 7;4:175. doi: 10.1038/s44172-025-00507-1 (PMC12504710; doi:10.1038/s44172-025-00507-1)
Supplement: Supplementary file 2 — Supplementary Information [file 44172_2025_507_MOESM2_ESM.pdf]

Supplementary File

# Optimising housing typology distributions for multi-hazard loss reductions in resource-constrained settings

Arvin Hadlos[1*], Aaron Opdyke[1], S.Ali Hadigheh[1]

[1]School of Civil Engineering, The University of Sydney, Sydney, Australia
[*]Corresponding author <arvin.hadlos@sydney.edu.au>

**Table of Contents**

1 Construction cost estimates....1
1.1 Activity-on-node diagrams....2
1.2 Bill of quantities....6
2 Pareto optimal solutions....16
3 References....20

## 1 Construction cost estimates

In this study, the construction cost for the typologies includes both material and labour costs. To estimate the material costs, we assumed a one-story five-meter by seven-meter (5m X 7m) housing configuration having a floor to top-of-beam height of 2.70 m. These assumptions conform to the prevailing characteristics of the typologies surveyed (via rapid visual assessment) in January 2023. Whereby required details for the estimates were not documented due to the limitation of direct visual observations, standard construction details were applied to derive the estimates. For example, we used the standard rebar spacing for residential projects (i.e., 0.40 m both ways for slabs/flooring). Conventional construction procedures were also assumed, such as the use of 1:2:4 proportion for cement-sand-gravel mixture typical for residential projects in the Philippines. We referred to the construction estimate guidelines by Fajardo [1] which provide detailed procedures for estimating construction material quantities applicable to the Philippine context. For the material prices, these were sourced from hardware stores in Itbayat representative of the actual prevailing market prices as of March 2024.

Meanwhile, to derive the labour costs, we projected the construction duration for each typology using activity-on-node diagrams (see Figure S1 to S6) considering a labour workforce of three skilled and three unskilled workers. We then used the standard construction wage in Itbayat (as reported by some construction workers as of March 2024) to inform the total labour cost based on the estimated construction duration for each typology. The resulting bill of quantities based on material and labour costs are shown in Table S1 to S6.

Abbreviations and notations

| | |
|---|---|
| ” | inches |
| CGI | corrugated galvanised iron |
| FTB | footing tie beam |
| GI | galvanised iron |
| kg. | kilogram |
| pcs. | pieces |
| RC | reinforced concrete |

## *1.1 Activity-on-node diagrams*

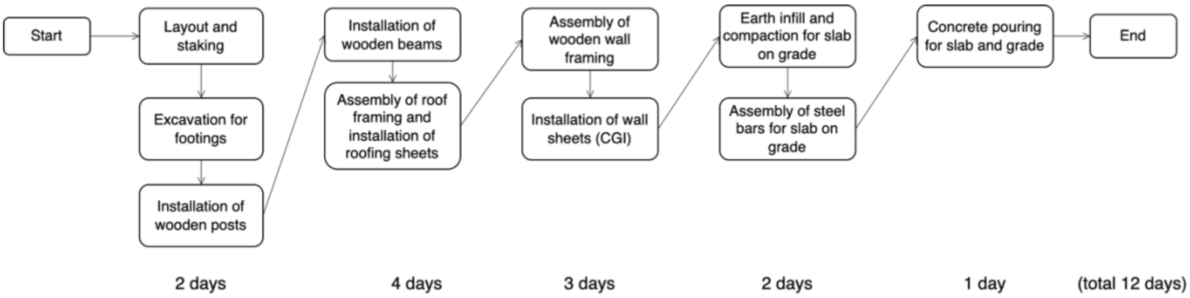


**Figure S1.** Activity-on-node diagram for LW-A.

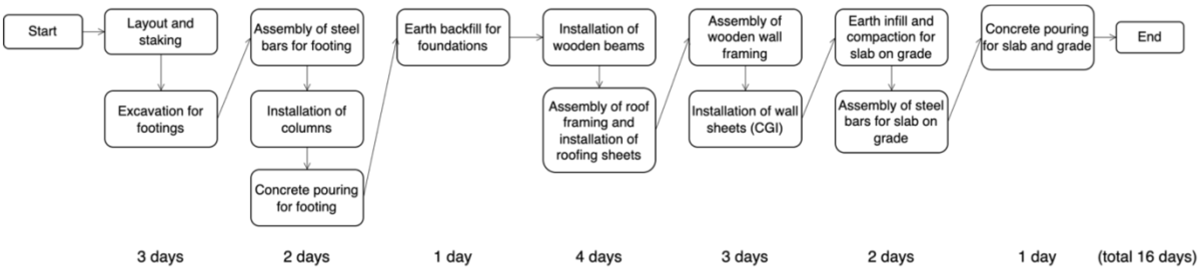


**Figure S2.** Activity-on-node diagram for LW-B.

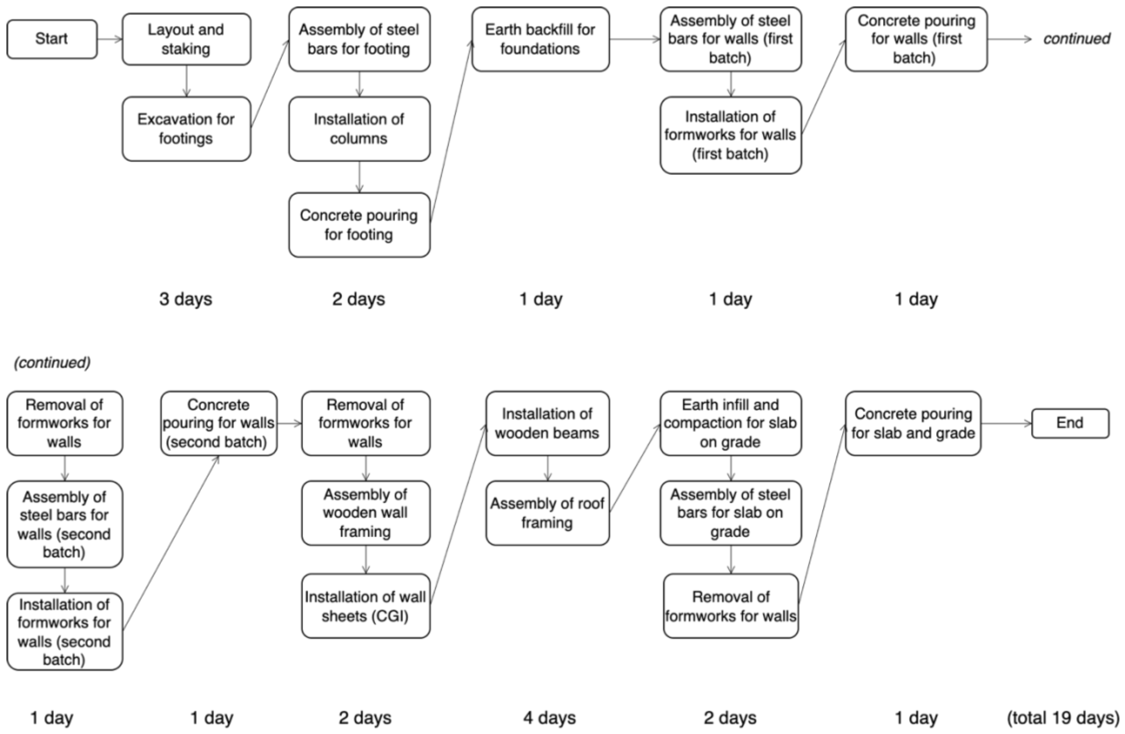


**Figure S3.** Activity-on-node diagram for SC-A.

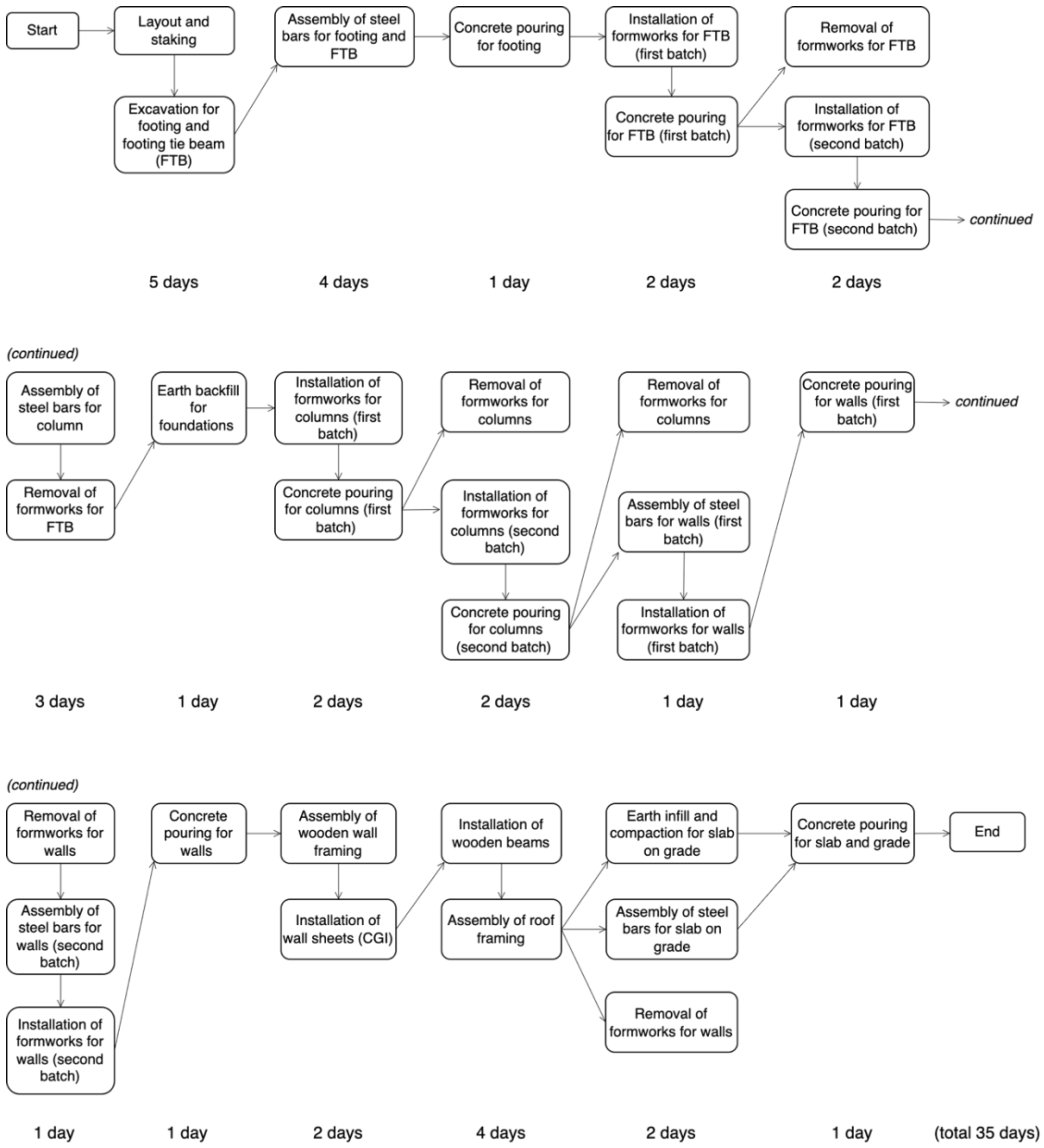


**Figure S4.** Activity-on-node diagram for SC-B.

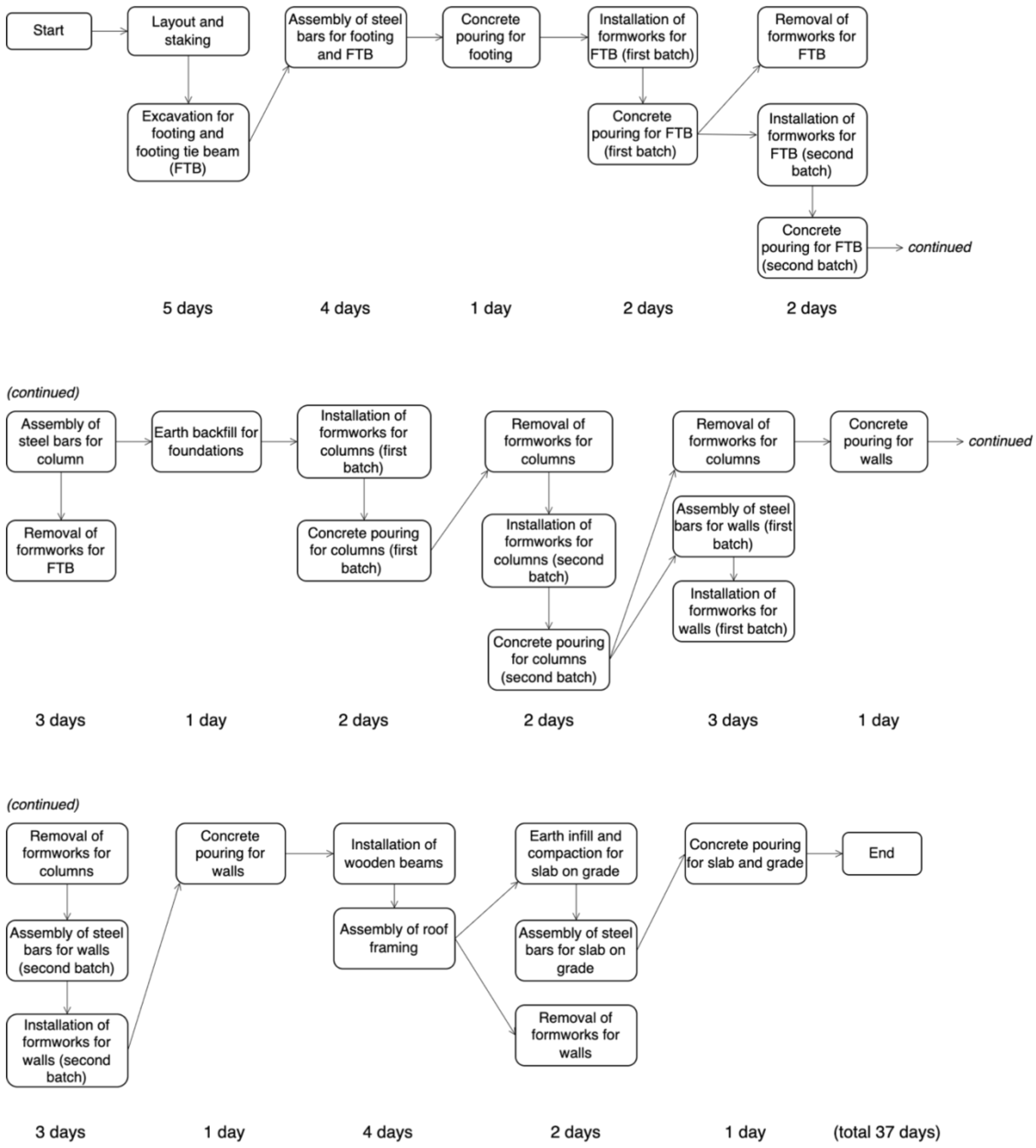


**Figure S5.** Activity-on-node diagram for RC-A.

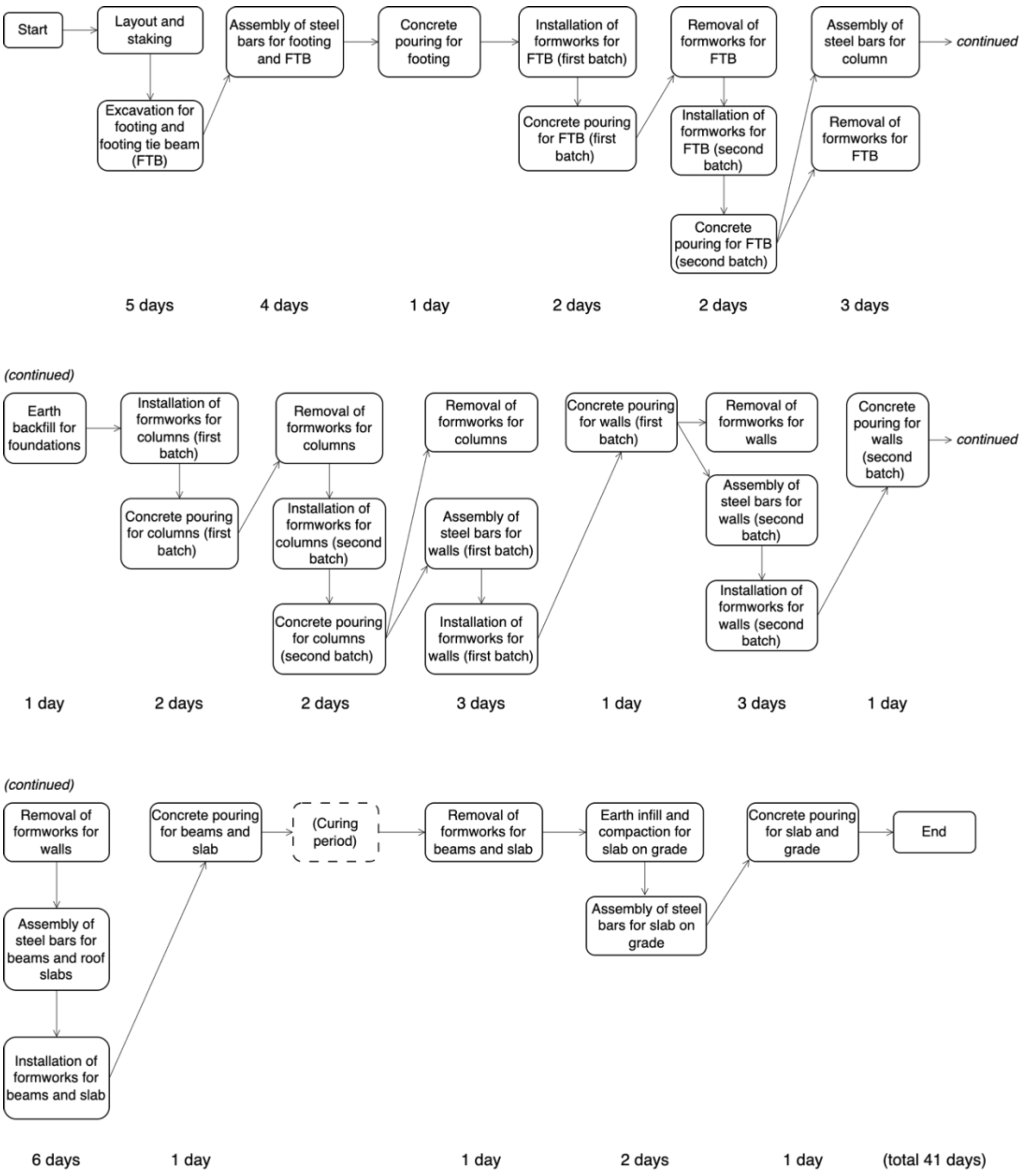


**Figure S6.** Activity-on-node diagram for RC-B.

## *1.2 Bill of quantities*

| **Construction cost estimates for LW-A (lightweight with wooden posts)** | | | | |
|---|---|---|---|---|
| *Summary* | | | | |
| Total cost (₱): | **294,255.00** | | | |
| Cost per square metre (₱): | **8,407.29** | | | |
| | | | | |
| **Detailed breakdown** | | | | |
| | **quantity** | **unit** | **unit price (₱)** | **total cost (₱)** |
| **Lateral load-resisting system** | | | | |

| | | | | |
|---|---|---|---|---|
| timber posts (4"X4") | 8 | pcs. | 810 | 6,480.00 |
| timber beams (2"X4") | 10 | pcs. | 550 | 5,500.00 |
| 5" common wire nail | 1 | kg. | 120 | 120.00 |
| | | | | |
| **Roof framing** | | | | |
| timber rafter (2"X4") | 22 | pcs. | 550 | 12,100.00 |
| timber purlins (2"X2") | 33 | pcs. | 415 | 13,695.00 |
| fascia board (2"X6") | 9 | pcs. | 700 | 6,300.00 |
| 5" common wire nail | 10 | kg. | 120 | 1,200.00 |
| | | | | |
| **Wall framing** | | | | |
| 2”X4” wood | 49 | pcs. | 550 | 26,950.00 |
| 5" common wire nail | 3.25 | kg. | 120 | 390.00 |
| | | | | |
| **Wall envelope** | | | | |
| Gauge 24 CGI sheet (3 metres) | 36 | pcs. | 1,300 | 46,800.00 |
| 5" common wire nail | 17.75 | kg. | 120 | 2,130.00 |
| | | | | |
| **Roof envelope** | | | | |
| Gauge 24 CGI sheet (3 metres) | 30 | pcs. | 1,300 | 39,000.00 |
| Plain GI | 3 | pcs. | 2,600 | 7,800.00 |
| 5" common wire nail | 14.75 | kg. | 120 | 1,770.00 |
| | | | | |
| **Flooring** | | | | |
| 40 kgs. cement | 32 | bags | 550 | 17,600.00 |
| sand | 1.75 | cu.m. | 8,000 | 14,000.00 |
| 3/4" gravel | 3.5 | cu.m. | 7,000 | 24,500.00 |
| 10 mm diameter steel bars | 75 | pcs. | 280 | 21,000.00 |
| GI tie wire | 1 | kg. | 120 | 120.00 |
| | | | | |
| **Labour** | | | | |
| foreman | 12 | days | 700 | 8,400.00 |
| skilled | 12 | days | 700 | 8,400.00 |
| skilled | 12 | days | 700 | 8,400.00 |
| unskilled (helper) | 12 | days | 600 | 7,200.00 |
| unskilled (helper) | 12 | days | 600 | 7,200.00 |
| unskilled (helper) | 12 | days | 600 | 7,200.00 |
| | | | | |
| | | | **Grand total** | **294,255.00** |

**Table S1.** Bill of quantities for LW-A (lightweight with wooden posts).

| Construction cost estimates for LW-B (lightweight with steel posts) | | | | |
|---|---|---|---|---|
| *Summary* | | | | |
| Total cost (₱): | **361,495.00** | | | |
| Cost per square metre (₱): | **10,328.43** | | | |
| | | | | |
| **Detailed breakdown** | | | | |
| | **quantity** | **unit** | **unit price (₱)** | **total cost (₱)** |
| **Lateral load-resisting system** | | | | |
| 5" diameter steel pipes | 4 | pcs. | 6,450 | 25,800.00 |
| timber beams (2"X4") | 10 | pcs. | 550 | 5,500.00 |
| 40 kgs. cement | 14 | bags | 550 | 7,700.00 |
| sand | 0.75 | cu.m. | 8,000 | 6,000.00 |
| 3/4" gravel | 1.5 | cu.m. | 7,000 | 10,500.00 |
| 16 mm diameter steel bars | 10 | pcs. | 800 | 8,000.00 |
| 5" common wire nail | 1 | kg. | 120 | 120.00 |
| GI tie wire | 1 | kg. | 120 | 120.00 |
| | | | | |
| **Roof framing** | | | | |
| timber rafter (2”X4”) | 22 | pcs. | 550 | 12,100.00 |
| timber purlins (2”X2”) | 33 | pcs. | 415 | 13,695.00 |
| fascia board (2”X6”) | 9 | pcs. | 700 | 6,300.00 |
| 5" common wire nail | 10 | kg. | 120 | 1,200.00 |
| | | | | |
| **Wall framing** | | | | |
| 2”X4” wood | 49 | pcs. | 550 | 26,950.00 |
| 5" common wire nail | 3.25 | kg. | 120 | 390.00 |
| | | | | |
| **Wall envelope** | | | | |
| Gauge 24 CGI sheet (3 metres) | 36 | pcs. | 1,300 | 46,800.00 |
| 5" common wire nail | 17.75 | kg. | 120 | 2,130.00 |
| | | | | |
| **Roof envelope** | | | | |
| Gauge 24 CGI sheet (3 metres) | 30 | pcs. | 1,300 | 39,000.00 |
| Plain GI | 3 | pcs. | 2,600 | 7,800.00 |
| 5" common wire nail | 14.75 | kg. | 120 | 1,770.00 |
| | | | | |
| **Flooring** | | | | |
| 40 kgs. cement | 32 | bags | 550 | 17,600.00 |
| sand | 1.75 | cu.m. | 8,000 | 14,000.00 |
| 3/4" gravel | 3.5 | cu.m. | 7,000 | 24,500.00 |
| 10 mm diameter steel bars | 75 | pcs. | 280 | 21,000.00 |
| GI tie wire | 1 | kg. | 120 | 120.00 |

| | | | | |
|---|---|---|---|---|
| | | | | |
| **Labour** | | | | |
| foreman | 16 | days | 700 | 11,200.00 |
| skilled | 16 | days | 700 | 11,200.00 |
| skilled | 16 | days | 700 | 11,200.00 |
| unskilled (helper) | 16 | days | 600 | 9,600.00 |
| unskilled (helper) | 16 | days | 600 | 9,600.00 |
| unskilled (helper) | 16 | days | 600 | 9,600.00 |
| | | | | |
| | | | **Grand total** | **361,495.00** |

**Table S2.** Bill of quantities for LW-B (lightweight with steel posts).

| **Construction cost estimates for SC-A (semi-concrete with steel posts)** | | | | |
|---|---|---|---|---|
| *Summary* | | | | |
| Total cost (₱): | **421,945.00** | | | |
| Cost per square metre (₱): | **12,055.57** | | | |
| | | | | |
| **Detailed breakdown** | | | | |
| | **quantity** | **unit** | **unit price (₱)** | **total cost (₱)** |
| **Lateral load-resisting system** | | | | |
| 5" diameter steel pipes | 4 | pcs. | 6,450 | 25,800.00 |
| timber beams (2”X4”) | 10 | pcs. | 550 | 5,500.00 |
| 40 kgs. cement | 14 | bags | 550 | 7,700.00 |
| sand | 0.75 | cu.m. | 8,000 | 6,000.00 |
| 3/4" gravel | 1.5 | cu.m. | 7,000 | 10,500.00 |
| 16 mm diameter steel bars | 10 | pcs. | 800 | 8,000.00 |
| 5" common wire nail | 1 | kg. | 120 | 120.00 |
| GI tie wire | 1 | kg. | 120 | 120.00 |
| | | | | |
| **Roof framing** | | | | |
| timber rafter (2”X4”) | 22 | pcs. | 550 | 12,100.00 |
| timber purlins (2”X2”) | 33 | pcs. | 415 | 13,695.00 |
| fascia board (2”X6”) | 9 | pcs. | 700 | 6,300.00 |
| 5" common wire nail | 10 | kg. | 120 | 1,200.00 |
| | | | | |
| **Wall framing** | | | | |
| 2”X4” wood frame | 35 | pcs. | 550 | 19,250.00 |
| 40 kgs. cement | 22 | bags | 550 | 12,100.00 |
| sand | 1.2 | cu.m. | 8,000 | 9,600.00 |
| 3/4" gravel | 2.4 | cu.m. | 7,000 | 16,800.00 |

| | | | | |
|---|---|---|---|---|
| 10 mm diameter steel bars | 23 | pcs. | 280 | 6,440.00 |
| 5" common wire nail | 2.25 | kg. | 120 | 270.00 |
| GI tie wire | 1 | kg. | 120 | 120.00 |
| | | | | |
| **Wall envelope** | | | | |
| Gauge 24 CGI sheet (3 metres) | 22 | pcs. | 1,300 | 28,600.00 |
| 5" common wire nail | 11 | kg. | 120 | 1,320.00 |
| | | | | |
| **Roof envelope** | | | | |
| Gauge 24 CGI sheet (3 metres) | 30 | pcs. | 1,300 | 39,000.00 |
| Plain GI | 3 | pcs. | 2,600 | 7,800.00 |
| 5" common wire nail | 14.75 | kg. | 120 | 1,770.00 |
| | | | | |
| **Flooring** | | | | |
| 40 kgs. cement | 32 | bags | 550 | 17,600.00 |
| sand | 1.75 | cu.m. | 8,000 | 14,000.00 |
| 3/4" gravel | 3.5 | cu.m. | 7,000 | 24,500.00 |
| 10 mm diameter steel bars | 75 | pcs. | 280 | 21,000.00 |
| GI tie wire | 1 | kg. | 120 | 120.00 |
| | | | | |
| **Formworks** | | | | |
| 2" X 2" wood | 34 | pcs. | 415 | 14,110.00 |
| 1/2" fibre cement board | 9 | pcs. | 1,800 | 16,200.00 |
| 2" common wire nail | 2 | kg. | 105 | 210.00 |
| | | | | |
| **Labour** | | | | |
| foreman | 19 | days | 700 | 13,300.00 |
| skilled | 19 | days | 700 | 13,300.00 |
| skilled | 19 | days | 700 | 13,300.00 |
| unskilled (helper) | 19 | days | 600 | 11,400.00 |
| unskilled (helper) | 19 | days | 600 | 11,400.00 |
| unskilled (helper) | 19 | days | 600 | 11,400.00 |
| | | | | |
| | | | **Grand total** | **421,945.00** |

**Table S3.** Bill of quantities for SC-A (semi-concrete with steel posts).

| **Construction cost estimates for SC-B (semi-concrete with RC posts)** | | | | |
|---|---|---|---|---|
| *Summary* | | | | |
| Total cost (₱): | **627,755.00** | | | |
| Cost per square metre (₱): | **17,935.86** | | | |

| | | | | |
|---|---|---|---|---|
| **Detailed breakdown** | | | | |
| | **quantity** | **unit** | **unit price (₱)** | **total cost (₱)** |
| **Lateral load-resisting system** | | | | |
| timber beams (2”X4”) | 10 | pcs. | 550 | 5,500.00 |
| 40 kgs. cement | 48 | bags | 550 | 26,400.00 |
| sand | 2.61 | cu.m. | 8,000 | 20,880.00 |
| 3/4" gravel | 5.22 | cu.m. | 7,000 | 36,540.00 |
| 16 mm diameter steel bars | 62 | pcs. | 800 | 49,600.00 |
| 10 mm diameter steel bars | 57 | pcs. | 280 | 15,960.00 |
| 5" common wire nail | 1 | kg. | 120 | 120.00 |
| GI tie wire | 1 | kg. | 120 | 120.00 |
| | | | | |
| **Roof framing** | | | | |
| timber rafter (2”X4”) | 22 | pcs. | 550 | 12,100.00 |
| timber purlins (2”X2”) | 33 | pcs. | 415 | 13,695.00 |
| fascia board (2”X6”) | 9 | pcs. | 700 | 6,300.00 |
| 5" common wire nail | 10 | kg. | 120 | 1,200.00 |
| | | | | |
| **Wall framing** | | | | |
| 2”X4” wood frame | 35 | pcs. | 550 | 19,250.00 |
| 40 kgs. cement | 22 | bags | 550 | 12,100.00 |
| sand | 1.2 | cu.m. | 8,000 | 9,600.00 |
| 3/4" gravel | 2.4 | cu.m. | 7,000 | 16,800.00 |
| 10 mm diameter steel bars | 23 | pcs. | 280 | 6,440.00 |
| 5" common wire nail | 2.25 | kg. | 120 | 270.00 |
| GI tie wire | 1 | kg. | 120 | 120.00 |
| | | | | |
| **Wall envelope** | | | | |
| Gauge 24 CGI sheet (3 metres) | 22 | pcs. | 1,300 | 28,600.00 |
| 5" common wire nail | 11 | kg. | 120 | 1,320.00 |
| | | | | |
| **Roof envelope** | | | | |
| Gauge 24 CGI sheet (3 metres) | 30 | pcs. | 1,300 | 39,000.00 |
| Plain GI | 3 | pcs. | 2,600 | 7,800.00 |
| 5" common wire nail | 14.75 | kg. | 120 | 1,770.00 |
| | | | | |
| **Flooring** | | | | |
| 40 kgs. cement | 32 | bags | 550 | 17,600.00 |
| sand | 1.75 | cu.m. | 8,000 | 14,000.00 |
| 3/4" gravel | 3.5 | cu.m. | 7,000 | 24,500.00 |
| 10 mm diameter steel bars | 75 | pcs. | 280 | 21,000.00 |

| | | | | |
|---|---|---|---|---|
| GI tie wire | 1 | kg. | 120 | 120.00 |
| | | | | |
| **Formworks** | | | | |
| 2" X 2" wood | 116 | pcs. | 415 | 48,140.00 |
| 1/2" fibre cement board | 19 | pcs. | 1,800 | 34,200.00 |
| 2" common wire nail | 2 | kg. | 105 | 210.00 |
| | | | | |
| **Labour** | | | | |
| foreman | 35 | days | 700 | 24,500.00 |
| skilled | 35 | days | 700 | 24,500.00 |
| skilled | 35 | days | 700 | 24,500.00 |
| unskilled (helper) | 35 | days | 600 | 21,000.00 |
| unskilled (helper) | 35 | days | 600 | 21,000.00 |
| unskilled (helper) | 35 | days | 600 | 21,000.00 |
| | | | | |
| | | | **Grand total** | **627,755.00** |

**Table S4**. Bill of quantities for SC-B (semi-concrete with RC posts).

| **Construction cost estimates for RC-A (reinforced concrete with lightweight roof)** | | | | |
|---|---|---|---|---|
| *Summary* | | | | |
| Total cost (₱): | **677,725.00** | | | |
| Cost per square metre (₱): | **19,363.57** | | | |
| | | | | |
| **Detailed breakdown** | | | | |
| | **quantity** | **unit** | **unit price (₱)** | **total cost (₱)** |
| **Lateral load-resisting system** | | | | |
| timber beams (2”X4”) | 10 | pcs. | 550 | 5,500.00 |
| 40 kgs. cement | 48 | bags | 550 | 26,400.00 |
| sand | 2.61 | cu.m. | 8,000 | 20,880.00 |
| 3/4" gravel | 5.22 | cu.m. | 7,000 | 36,540.00 |
| 16 mm diameter steel bars | 62 | pcs. | 800 | 49,600.00 |
| 10 mm diameter steel bars | 57 | pcs. | 280 | 15,960.00 |
| 5" common wire nail | 1 | kg. | 120 | 120.00 |
| GI tie wire | 1 | kg. | 120 | 120.00 |
| | | | | |
| **Roof framing** | | | | |
| timber rafter (2”X4”) | 22 | pcs. | 550 | 12,100.00 |
| timber purlins (2”X2”) | 33 | pcs. | 415 | 13,695.00 |
| fascia board (2”X6”) | 9 | pcs. | 700 | 6,300.00 |
| 5" common wire nail | 10 | kg. | 120 | 1,200.00 |

| | | | | |
|---|---|---|---|---|
| | | | | |
| **Wall framing/envelope** | | | | |
| 40 kgs. cement | 52 | bags | 550 | 28,600.00 |
| sand | 2.84 | cu.m. | 8,000 | 22,720.00 |
| 3/4" gravel | 5.68 | cu.m. | 7,000 | 39,760.00 |
| 10 mm diameter steel bars | 49 | pcs. | 280 | 13,720.00 |
| GI tie wire | 2 | kg. | 120 | 240.00 |
| | | | | |
| **Roof envelope** | | | | |
| Gauge 24 CGI sheet (3 metres) | 30 | pcs. | 1,300 | 39,000.00 |
| Plain GI | 3 | pcs. | 2,600 | 7,800.00 |
| 5" common wire nail | 14.75 | kg. | 120 | 1,770.00 |
| | | | | |
| **Flooring** | | | | |
| 40 kgs. cement | 32 | bags | 550 | 17,600.00 |
| sand | 1.75 | cu.m. | 8,000 | 14,000.00 |
| 3/4" gravel | 3.5 | cu.m. | 7,000 | 24,500.00 |
| 10 mm diameter steel bars | 75 | pcs. | 280 | 21,000.00 |
| GI tie wire | 1 | kg. | 120 | 120.00 |
| | | | | |
| **Formworks** | | | | |
| 2" X 2" wood | 144 | pcs. | 415 | 59,760.00 |
| 1/2" fibre cement board | 30 | pcs. | 1,800 | 54,000.00 |
| 2" common wire nail | 4 | kg. | 105 | 420.00 |
| | | | | |
| **Labour** | | | | |
| foreman | 37 | days | 700 | 25,900.00 |
| skilled | 37 | days | 700 | 25,900.00 |
| skilled | 37 | days | 700 | 25,900.00 |
| unskilled (helper) | 37 | days | 600 | 22,200.00 |
| unskilled (helper) | 37 | days | 600 | 22,200.00 |
| unskilled (helper) | 37 | days | 600 | 22,200.00 |
| | | | | |
| | | | **Grand total** | **677,725.00** |

**Table S5**. Bill of quantities for RC-A (reinforced concrete with lightweight roof).

| **Construction cost estimates for RC-B (reinforced concrete with slab roof)** | | | | |
|---|---|---|---|---|
| *Summary* | | | | |
| Total cost (₱): | **881,395.00** | | | |
| Cost per square metre (₱): | **25,182.71** | | | |

| | | | | |
|---|---|---|---|---|
| | | | | |
| **Detailed breakdown** | | | | |
| | **quantity** | **unit** | **unit price (₱)** | **total cost (₱)** |
| **Lateral load-resisting system** | | | | |
| 40 kgs. cement | 64 | bags | 550 | 35,200.00 |
| sand | 3.48 | cu.m. | 8,000 | 27,840.00 |
| 3/4" gravel | 6.96 | cu.m. | 7,000 | 48,720.00 |
| 16 mm diameter steel bars | 90 | pcs. | 800 | 72,000.00 |
| 10 mm diameter steel bars | 87 | pcs. | 280 | 24,360.00 |
| GI tie wire | 1 | kg. | 120 | 120.00 |
| | | | | |
| **Roof framing/envelope** | | | | |
| 40 kgs. cement | 57 | bags | 550 | 31,350.00 |
| sand | 3.15 | cu.m. | 8,000 | 25,200.00 |
| 3/4" gravel | 6.3 | cu.m. | 7,000 | 44,100.00 |
| 10 mm diameter steel bars | 78 | pcs. | 280 | 21,840.00 |
| GI tie wire | 1 | kg. | 120 | 120.00 |
| | | | | |
| **Wall framing/envelope** | | | | |
| 40 kgs. cement | 46 | bags | 550 | 25,300.00 |
| sand | 2.52 | cu.m. | 8,000 | 20,160.00 |
| 3/4" gravel | 5.04 | cu.m. | 7,000 | 35,280.00 |
| 10 mm diameter steel bars | 43 | pcs. | 280 | 12,040.00 |
| GI tie wire | 2 | kg. | 120 | 240.00 |
| | | | | |
| **Flooring** | | | | |
| 40 kgs. cement | 32 | bags | 550 | 17,600.00 |
| sand | 1.75 | cu.m. | 8,000 | 14,000.00 |
| 3/4" gravel | 3.5 | cu.m. | 7,000 | 24,500.00 |
| 10 mm diameter steel bars | 75 | pcs. | 280 | 21,000.00 |
| GI tie wire | 1 | kg. | 120 | 120.00 |
| | | | | |
| **Formworks** | | | | |
| 2" X 2" wood | 265 | pcs. | 415 | 109,975.00 |
| 1/2" fibre cement board | 61 | pcs. | 1,800 | 109,800.00 |
| 2" common wire nail | 6 | kg. | 105 | 630.00 |
| | | | | |
| **Labour** | | | | |
| foreman | 41 | days | 700 | 28,700.00 |
| skilled | 41 | days | 700 | 28,700.00 |
| skilled | 41 | days | 700 | 28,700.00 |
| unskilled (helper) | 41 | days | 600 | 24,600.00 |

| | | | | |
|---|---|---|---|---|
| unskilled (helper) | 41 | days | 600 | 24,600.00 |
| unskilled (helper) | 41 | days | 600 | 24,600.00 |
| | | | | |
| | | | **Grand total** | **881,395.00** |

**Table S6**. Bill of quantities for RC-B (reinforced concrete with slab roof).

## 2 Pareto optimal solutions

| Scenario # | Direct economic losses (in PHP) | | Scores for Pareto optimal ranking | | | Building stock distribution | | | | | |
|---|---|---|---|---|---|---|---|---|---|---|---|
| | PEIS VII | 270 km/h | PEIS VII | 270 km/h | Composite score | LWA | LWB | SCA | SCB | RCA | RCB |
| 11628 | 330,643,889 | 194,809,277 | 0.005673246 | 0.02700054 | 0.032673786 | 5% | 5% | 5% | 5% | 5% | 75% |
| 15 | 161,719,217 | 385,296,870 | 0.02700054 | 0.005673246 | 0.032673786 | 5% | 75% | 5% | 5% | 5% | 5% |
| 11623 | 319,468,398 | 205,180,265 | 0.005691644 | 0.01800036 | 0.023692004 | 10% | 5% | 5% | 5% | 5% | 70% |
| 14 | 162,130,218 | 382,028,657 | 0.01800036 | 0.005691644 | 0.023692004 | 10% | 70% | 5% | 5% | 5% | 5% |
| 11624 | 318,852,908 | 208,356,401 | 0.005710453 | 0.014727567 | 0.02043802 | 5% | 10% | 5% | 5% | 5% | 70% |
| 13 | 162,723,840 | 378,799,591 | 0.014727567 | 0.005710453 | 0.02043802 | 15% | 65% | 5% | 5% | 5% | 5% |
| 11608 | 308,051,879 | 216,001,788 | 0.005729688 | 0.012960259 | 0.018689947 | 15% | 5% | 5% | 5% | 5% | 65% |
| 12 | 163,333,090 | 375,785,701 | 0.012960259 | 0.005729688 | 0.018689947 | 20% | 60% | 5% | 5% | 5% | 5% |
| 11609 | 307,468,066 | 219,223,797 | 0.005749366 | 0.011825054 | 0.01757442 | 10% | 10% | 5% | 5% | 5% | 65% |
| 11 | 163,957,401 | 372,703,382 | 0.011825054 | 0.005749366 | 0.01757442 | 25% | 55% | 5% | 5% | 5% | 5% |
| 11610 | 306,868,700 | 222,332,970 | 0.005769506 | 0.011020629 | 0.016790135 | 5% | 15% | 5% | 5% | 5% | 65% |
| 10 | 164,672,020 | 369,770,738 | 0.011020629 | 0.005769506 | 0.016790135 | 30% | 50% | 5% | 5% | 5% | 5% |
| 11573 | 296,565,843 | 226,277,475 | 0.005790127 | 0.010413431 | 0.016203558 | 20% | 5% | 5% | 5% | 5% | 60% |
| 9 | 165,104,516 | 366,513,292 | 0.010413431 | 0.005790127 | 0.016203558 | 35% | 45% | 5% | 5% | 5% | 5% |
| 11574 | 295,778,659 | 229,245,878 | 0.005811249 | 0.009934496 | 0.015745744 | 15% | 10% | 5% | 5% | 5% | 60% |
| 8 | 165,873,869 | 363,660,877 | 0.009934496 | 0.005811249 | 0.015745744 | 40% | 40% | 5% | 5% | 5% | 5% |
| 7 | 166,292,994 | 360,397,559 | 0.009544306 | 0.005832893 | 0.0153772 | 45% | 35% | 5% | 5% | 5% | 5% |
| 11575 | 295,182,952 | 232,435,456 | 0.005832893 | 0.009544306 | 0.0153772 | 10% | 15% | 5% | 5% | 5% | 60% |
| 6 | 167,038,511 | 357,491,529 | 0.009218448 | 0.005855084 | 0.015073531 | 50% | 30% | 5% | 5% | 5% | 5% |
| 11576 | 294,796,288 | 235,696,945 | 0.005855084 | 0.009218448 | 0.015073531 | 5% | 20% | 5% | 5% | 5% | 60% |
| 5 | 167,472,726 | 354,198,684 | 0.008940939 | 0.005877845 | 0.014818784 | 55% | 25% | 5% | 5% | 5% | 5% |
| 11503 | 285,064,184 | 236,938,396 | 0.005877845 | 0.008940939 | 0.014818784 | 25% | 5% | 5% | 5% | 5% | 55% |

| | | | | | | | | | | | |
|---|---|---|---|---|---|---|---|---|---|---|---|
| 4 | 168,071,741 | 351,051,812 | 0.00870084 | 0.005901202 | 0.014602042 | 60% | 20% | 5% | 5% | 5% | 5% |
| 11504 | 284,437,175 | 240,075,052 | 0.005901202 | 0.00870084 | 0.014602042 | 20% | 10% | 5% | 5% | 5% | 55% |
| 3 | 168,668,524 | 348,016,025 | 0.008490379 | 0.005925184 | 0.014415562 | 65% | 15% | 5% | 5% | 5% | 5% |
| 11505 | 283,745,624 | 242,936,879 | 0.005925184 | 0.008490379 | 0.014415562 | 15% | 15% | 5% | 5% | 5% | 55% |
| 2 | 169,246,530 | 344,963,082 | 0.008303867 | 0.005949819 | 0.014253686 | 70% | 10% | 5% | 5% | 5% | 5% |
| 11506 | 283,288,837 | 246,252,129 | 0.005949819 | 0.008303867 | 0.014253686 | 10% | 20% | 5% | 5% | 5% | 55% |
| 1 | 169,982,214 | 342,009,468 | 0.008137033 | 0.00597514 | 0.014112173 | 75% | 5% | 5% | 5% | 5% | 5% |
| 11377 | 273,557,925 | 247,624,032 | 0.00597514 | 0.008137033 | 0.014112173 | 30% | 5% | 5% | 5% | 5% | 50% |
| 3064 | 179,706,197 | 340,867,610 | 0.007986603 | 0.00600118 | 0.013987783 | 55% | 20% | 5% | 5% | 5% | 10% |
| 11378 | 272,918,403 | 250,754,293 | 0.00600118 | 0.007986603 | 0.013987783 | 25% | 10% | 5% | 5% | 5% | 50% |
| 3063 | 180,168,810 | 337,549,713 | 0.007850015 | 0.006027976 | 0.013877991 | 60% | 15% | 5% | 5% | 5% | 10% |
| 11379 | 272,320,370 | 253,891,151 | 0.006027976 | 0.007850015 | 0.013877991 | 20% | 15% | 5% | 5% | 5% | 50% |
| 3062 | 180,863,829 | 334,652,767 | 0.007725238 | 0.006055566 | 0.013780803 | 65% | 10% | 5% | 5% | 5% | 10% |
| 11380 | 271,751,401 | 256,979,382 | 0.006055566 | 0.007725238 | 0.013780803 | 15% | 20% | 5% | 5% | 5% | 50% |
| 3061 | 181,453,337 | 331,501,319 | 0.007610632 | 0.006083993 | 0.013694625 | 70% | 5% | 5% | 5% | 5% | 10% |
| 11167 | 262,352,399 | 257,953,971 | 0.006083993 | 0.007610632 | 0.013694625 | 35% | 5% | 5% | 5% | 5% | 45% |
| 5444 | 191,440,238 | 330,220,789 | 0.007504862 | 0.006113301 | 0.013618163 | 50% | 20% | 5% | 5% | 5% | 15% |
| 11168 | 261,755,450 | 261,129,624 | 0.006113301 | 0.007504862 | 0.013618163 | 30% | 10% | 5% | 5% | 5% | 45% |
| 5443 | 191,882,978 | 326,940,752 | 0.007406827 | 0.00614354 | 0.013550367 | 55% | 15% | 5% | 5% | 5% | 15% |
| 11169 | 260,995,447 | 264,039,989 | 0.00614354 | 0.007406827 | 0.013550367 | 25% | 15% | 5% | 5% | 5% | 45% |
| 5442 | 192,461,239 | 323,821,432 | 0.007315607 | 0.006174762 | 0.013490369 | 60% | 10% | 5% | 5% | 5% | 15% |
| 11170 | 260,598,584 | 267,304,627 | 0.006174762 | 0.007315607 | 0.013490369 | 20% | 20% | 5% | 5% | 5% | 45% |
| 5441 | 193,225,689 | 320,931,695 | 0.007230432 | 0.006207023 | 0.013437454 | 65% | 5% | 5% | 5% | 5% | 15% |
| 10837 | 250,697,562 | 268,818,308 | 0.006207023 | 0.007230432 | 0.013437454 | 40% | 5% | 5% | 5% | 5% | 40% |
| 7264 | 202,962,510 | 319,945,851 | 0.007150646 | 0.006240385 | 0.013391031 | 45% | 20% | 5% | 5% | 5% | 20% |
| 10838 | 250,060,355 | 271,930,672 | 0.006240385 | 0.007150646 | 0.013391031 | 35% | 10% | 5% | 5% | 5% | 40% |
| 7263 | 203,536,208 | 316,856,009 | 0.007075691 | 0.006274915 | 0.013350606 | 50% | 15% | 5% | 5% | 5% | 20% |
| 10839 | 249,495,353 | 275,091,799 | 0.006274915 | 0.007075691 | 0.013350606 | 30% | 15% | 5% | 5% | 5% | 40% |
| 7262 | 204,124,781 | 313,836,584 | 0.007005086 | 0.006310686 | 0.013315771 | 55% | 10% | 5% | 5% | 5% | 20% |

| | | | | | | | | | | | |
|---|---|---|---|---|---|---|---|---|---|---|---|
| 10840 | 248,900,698 | 278,175,787 | 0.006310686 | 0.007005086 | 0.013315771 | 25% | 20% | 5% | 5% | 5% | 40% |
| 7261 | 204,701,301 | 310,576,712 | 0.006938415 | 0.006347776 | 0.013286191 | 60% | 5% | 5% | 5% | 5% | 20% |
| 10342 | 239,430,991 | 279,231,186 | 0.006347776 | 0.006938415 | 0.013286191 | 45% | 5% | 5% | 5% | 5% | 35% |
| 8629 | 214,536,724 | 309,266,738 | 0.006875316 | 0.006386274 | 0.013261589 | 40% | 20% | 5% | 5% | 5% | 25% |
| 10343 | 238,842,242 | 282,281,368 | 0.006386274 | 0.006875316 | 0.013261589 | 40% | 10% | 5% | 5% | 5% | 35% |
| 8628 | 214,929,525 | 306,087,444 | 0.006815472 | 0.006426273 | 0.013241745 | 45% | 15% | 5% | 5% | 5% | 25% |
| 10344 | 238,113,201 | 285,296,294 | 0.006426273 | 0.006815472 | 0.013241745 | 35% | 15% | 5% | 5% | 5% | 35% |
| 8627 | 215,726,723 | 303,119,163 | 0.006758605 | 0.006467878 | 0.013226483 | 50% | 10% | 5% | 5% | 5% | 25% |
| 10345 | 237,651,045 | 288,516,143 | 0.006467878 | 0.006758605 | 0.013226483 | 30% | 20% | 5% | 5% | 5% | 35% |
| 8626 | 216,299,312 | 300,007,584 | 0.006704469 | 0.006511204 | 0.013215673 | 55% | 5% | 5% | 5% | 5% | 25% |
| 9627 | 227,719,722 | 289,836,563 | 0.006511204 | 0.006704469 | 0.013215673 | 50% | 5% | 5% | 5% | 5% | 30% |
| 9628 | 227,081,804 | 292,913,126 | 0.006556378 | 0.006652845 | 0.013209223 | 45% | 10% | 5% | 5% | 5% | 30% |
| 9630 | 225,900,540 | 299,076,682 | 0.006652845 | 0.006556378 | 0.013209223 | 35% | 20% | 5% | 5% | 5% | 30% |
| 9629 | 226,479,311 | 296,019,937 | 0.006603539 | 0.006603539 | 0.013207079 | 40% | 15% | 5% | 5% | 5% | 30% |

**Table S7**. Pareto optimal solutions for Case 1 (PEIS VII & 270 km/h).

| **Scenario #** | **Direct economic loss (in PHP)** | | **Scores for Pareto optimal ranking** | | | **Building stock distribution** | | | | | |
|---|---|---|---|---|---|---|---|---|---|---|---|
| | **PEIS VIII** | **300 km/h** | **PEIS VIII** | **300 km/h** | **Composite score** | **LWA** | **LWB** | **SCA** | **SCB** | **RCA** | **RCB** |
| 11628 | 629,185,361 | 331,094,944 | 0.022957675 | 0.076178823 | 0.099136498 | 5% | 5% | 5% | 5% | 5% | 75% |
| 1 | 310,680,271 | 403,105,449 | 0.076178823 | 0.022957675 | 0.099136498 | 75% | 5% | 5% | 5% | 5% | 5% |
| 11623 | 606,842,199 | 336,037,316 | 0.023428376 | 0.050785882 | 0.074214258 | 10% | 5% | 5% | 5% | 5% | 70% |
| 3061 | 333,613,205 | 397,793,439 | 0.050785882 | 0.023428376 | 0.074214258 | 70% | 5% | 5% | 5% | 5% | 10% |
| 11608 | 584,101,527 | 341,513,574 | 0.023954597 | 0.041552085 | 0.065506683 | 15% | 5% | 5% | 5% | 5% | 65% |
| 5441 | 356,828,476 | 392,520,890 | 0.041552085 | 0.023954597 | 0.065506683 | 65% | 5% | 5% | 5% | 5% | 15% |

| | | | | | | | | | | | |
|---|---|---|---|---|---|---|---|---|---|---|---|
| 11573 | 561,560,277 | 346,509,750 | 0.024548389 | 0.036565835 | 0.061114224 | 20% | 5% | 5% | 5% | 5% | 60% |
| 7261 | 379,581,182 | 387,564,025 | 0.036565835 | 0.024548389 | 0.061114224 | 60% | 5% | 5% | 5% | 5% | 20% |
| 11503 | 538,837,184 | 351,494,598 | 0.0252258 | 0.033362988 | 0.058588789 | 25% | 5% | 5% | 5% | 5% | 55% |
| 8626 | 402,568,525 | 382,454,594 | 0.033362988 | 0.0252258 | 0.058588789 | 55% | 5% | 5% | 5% | 5% | 25% |
| 9627 | 425,172,974 | 377,485,899 | 0.031093397 | 0.026008757 | 0.057102154 | 50% | 5% | 5% | 5% | 5% | 30% |
| 11377 | 515,893,516 | 356,979,231 | 0.026008757 | 0.031093397 | 0.057102154 | 30% | 5% | 5% | 5% | 5% | 50% |
| 10342 | 448,141,503 | 372,270,392 | 0.029380262 | 0.026928129 | 0.056308392 | 45% | 5% | 5% | 5% | 5% | 35% |
| 11167 | 493,479,698 | 361,862,506 | 0.026928129 | 0.029380262 | 0.056308392 | 35% | 5% | 5% | 5% | 5% | 45% |
| 10837 | 470,679,801 | 367,346,345 | 0.028029002 | 0.028029002 | 0.056058004 | 40% | 5% | 5% | 5% | 5% | 40% |

**Table S8**. Pareto optimal solutions for Case 2 (PEIS VIII & 300 km/h).

## 3 References

[1] M. Fajardo, Jr., *Simplified Construction Estimate*, 2000 Edition. 2000.
